# Vesicular stomatitis Indiana virus near-full-length genome sequences reveal low genetic diversity during the 2019 outbreak in Colorado, USA

**DOI:** 10.3389/fvets.2023.1110483

**Published:** 2023-02-14

**Authors:** Miranda R. Bertram, Case Rodgers, Kirsten Reed, Lauro Velazquez-Salinas, Angela Pelzel-McCluskey, Christie Mayo, Luis Rodriguez

**Affiliations:** ^1^Foreign Animal Disease Research Unit, Plum Island Animal Disease Center, Agricultural Research Service, United States Department of Agriculture, Greenport, NY, United States; ^2^Department of Microbiology, Immunology, and Pathology, Colorado State University, Fort Collins, CO, United States; ^3^Veterinary Services, Animal and Plant Health Inspection Service, United States Department of Agriculture, Fort Collins, CO, United States

**Keywords:** vesicular stomatitis Indiana virus, VSIV, 2019, USA, Colorado, VSV

## 1. Introduction

Vesicular stomatitis virus (VSV; *Vesiculovirus, Rhabdoviridae*) is the causative agent of vesicular stomatitis (VS), an economically important disease of livestock (1). Clinical signs are indistinguishable from foot-and-mouth disease and include vesicles on the muzzle, oral cavity, coronary band, udder, and prepuce (2). In the US, VS-like lesions require immediate reporting to state and federal animal health officials. Although the disease is usually self-limiting, animal movement restrictions and trade limitations result in significant economic losses during an outbreak (3).

VSV is a single-stranded RNA virus, with a ~11 kb genome encoding five proteins (N, P, M, G, L) (4). The two most common serotypes affecting domestic animals are New Jersey (VSNJV) and Indiana (VSIV) (2). VSNJV has a higher genetic diversity than VSIV in the field (5) and increased virulence in pathogenesis studies (6). However, both serotypes cause periodic outbreaks in the US (7).

Little is known about the multiple factors involved in the maintenance and transmission of VSV in endemic settings and in epidemic outbreaks, however a variety of insects have been implicated as vectors for VSV transmission (8). Insect vectors play an important role in the spread of VSV between premises, while direct contact between livestock may be an important mechanism of transmission within infected premises (8).

VSV is endemic in South and Central America, and in southern Mexico, where outbreaks occur seasonally (2). The virus periodically causes outbreaks in central and northern Mexico, and spreads into the US, causing outbreaks in the US on an approximately 5–8 year cycle (7, 9, 10). Recent studies indicate that epidemic lineages of VSNJV present different phenotypes than endemic lineages in both vertebrate and invertebrate hosts (11, 12). Epidemic lineages causing outbreaks in the US likely originate in endemic regions of Mexico and spread northward (7, 9). Following incursion into the US, the virus likely overwinters in an as yet unknown host(s) and reemerges to cause outbreaks again the following year (1, 13). The virus exhibits different population dynamics during incursion (first year of an outbreak) and expansion [subsequent year(s) of the outbreak] (14). Outbreaks in the US typically last 1–3 years (7, 10). The majority of outbreaks in the US have been caused by VSNJV. However, sporadic outbreaks caused by VSIV have been reported in the US, one during 1997–1998 and most recently during 2019–2020 (3). The outbreak in 2019 was the largest VS outbreak in the US in the past 40 years (3).

The 2019 US outbreak began in June 2019, and eventually affected 8 states (Texas, New Mexico, Colorado, Wyoming, Oklahoma, Nebraska, Utah, Kansas) (3). Colorado was the most-affected state during 2019, with 693 premises affected in 38 counties; the first case was identified in Colorado on 3 July 2019, and the last premises was released from quarantine on 17 December 2019 (3). The virus overwintered, and further outbreaks occurred during 2020 in New Mexico, Arizona, Texas, Kansas, Nebraska, Oklahoma, Missouri, and Arkansas. Interestingly, Colorado did not report any VS cases during 2020. Affected premises were quarantined and managed as previously described, however no additional measures were performed which might help explain the lack of cases in Colorado in 2020 (3).

The paucity of full-length VSV sequences, and particularly VSIV sequences, currently available in public databases limits understanding of the genetic basis associated with the emergence of epidemic lineages. The goal of this project was to produce a collection of VSIV genomes representing an epidemic lineage circulating in the US. We report herein 86 near-full-length genomes obtained from VSIV isolates collected from naturally infected horses in 27 counties of Colorado during 2019. The reader is directed to Pelzel-McCluskey et al. (3) for a detailed description of the outbreak.

## 2. Methods and analysis

### 2.1. Sample collection

The National Animal Health Laboratory Network (NAHLN) is a collaborative network of veterinary diagnostic laboratories that provide testing capacity for ongoing surveillance programs and respond quickly and effectively to disease outbreaks as they occur. In 2019, Colorado State University, a member of the NAHLN, was activated by the National Veterinary Services Laboratories (NVSL) to respond to the VSV outbreak in Colorado. The samples sequenced herein were tested as part of these efforts. Swabs of vesicular lesions were collected by veterinarians in Colorado and submitted for VSV diagnostic testing at the Colorado State University Diagnostic Laboratories between July and October 2019.

RNA was extracted from samples using an automated bead-based platform and tested by real-time reverse transcriptase polymerase chain reaction (rRT-PCR) in accordance with the NAHLN standard operating procedure. Extracted RNA was stored at −80°C after initial testing. In some instances, original samples that had been stored at −80°C were re-processed in an attempt to improve sequencing quality. RNA from these samples was extracted using the same NAHLN SOP as previously used during initial diagnostic testing.

### 2.2. Sequencing

Samples with resulting cycle threshold (C_t_) values of ≤26.99 upon initial rRT-PCR testing were selected for next generation sequencing. This cut-off was chosen based on results of a pilot study sequencing a subset of samples with Cts ranging from 16.09 to 33.29. RNA from the original extraction was sequenced in duplicate as previously described (14, 15). Briefly, DNA was depleted from extracted samples using the DNA-free DNase kit (Ambion, Austin, TX) following manufacturer's instructions. First-strand synthesis was performed using a Superscript™ II Reverse Transcriptase kit (Invitrogen, Carlsbad, CA) with random hexamer primers and one conserved VSV intergenic region specific primer (5). A NEBNext^®^ Ultra Non-Directional RNA Second Strand Synthesis Module was used to produce the second strand of cDNA. cDNA was purified using AmPure XP beads (Beckman-Coulter, Brea, CA), followed by the creation of cDNA libraries using a Nextera XT DNA Library Preparation kit. Next generation sequencing (NGS) was performed using an Illumina NextSeq550 instrument, with paired-end reads. Reads were quality trimmed and *de novo* assembled. Trimmed reads were also mapped to a VSIV reference genome [IN98COE, GenBank accession #AF473864; (16)]. Consensus sequences were extracted from each mapping and assembly for each sample then aligned, and a final consensus sequence was extracted from the alignment for each sample. All analyses were performed in CLC Workbench v21 using default parameters.

### 2.3. Analysis

A total of 86 near-full-length sequences were generated (Supplementary Table S1), representing 27 counties in Colorado. The 10,821–11,185 nucleotide (nt) genomes encode for 5 structural and non-structural proteins: nucleocapsid protein (1,269 nt), phosphoprotein (798 nt), matrix protein (690 nt), glycoprotein (1,536 nt), and L-polymerase (6,330 nt), with intergenic spacer regions separating each protein coding region. These sequences contain a 14–22 nt insertion in the G-L intergenic region compared to IN98COE. Interestingly, insertions in the G-L intergenic region have been noted in other VSIV lineages originating in Central America (16), suggesting this lineage may have also originated in that region. However, further research is needed to determine the origin of the causative lineage of the 2019–2020 VSIV outbreak in the US.

The 86 sequences generated in the current study were aligned with all publicly available sequences from the 2019–2020 VSIV outbreak in the US (*n* = 20), and IN98COE was included as an outgroup to investigate genetic variation within the outbreak. Sequences were aligned using MUSCLE (17) implemented in Geneious Prime v2022 (www.geneious.com). The Tamura 3-parameter model with uniform rates was identified as the most appropriate model based on Bayesian information criterion (BIC), and a phylogenetic tree was constructed using maximum likelihood implemented in Mega X (18). The final consensus tree was visualized using FigTree v1.4.4 (19).

**Figure 1:**
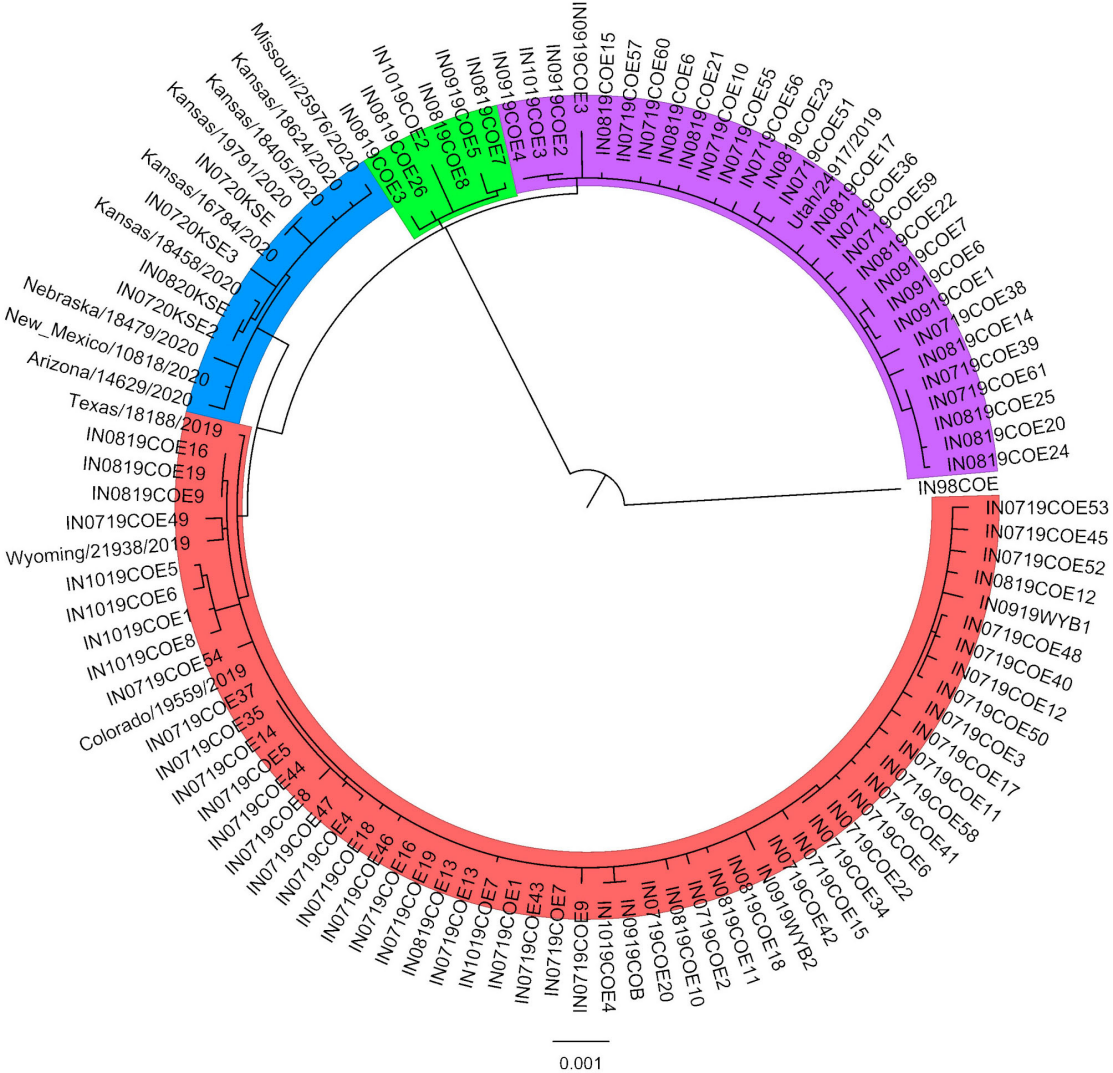
Maximum-likelihood phylogenetic analysis of the 2019–2020 VSIV outbreak in the US. A representative sequence from the 1997–1998 US outbreak (IN98COE) is included as an outgroup. Sequences from 2019 form three distinct groups (Group 1: red; Group 2: purple; Group 3: green), and sequences from 2020 form an additional distinct group (blue).

Phylogenetic analysis revealed three distinct groups within the 2019 sequences, and the 2020 sequences formed an additional distinct group (Figure 1). The 2019 sequences grouped by geographic area rather than time. Isolates collected at timepoints throughout the outbreak are represented in each of the 2019 groups. Isolates in group 1 were collected in counties in the northeast part of Colorado, while group 2 were collected in counties in the southwest, and group 3 were collected in the central part of the state. Within-group similarity was 99.93–99.96%, and between-group similarity was 99.80–99.90% (Table 1). Similar to patterns in previous outbreaks, the sequences from the expansion year of the outbreak (2020) form a monophyletic group within the sequences from the incursion year (2019) (14). Overall, there was low variation among isolates from the 2019–2020 outbreak, which is consistent with previous findings of low genetic variability in VSIV, especially in comparison with VSNJV (14, 20). Ongoing analyses of these sequences are investigating additional evolutionary, epidemiological, and ecological aspects of the outbreak. Similar to previous work with VSNJV (21), we are also developing an infectious clone of this epidemic VSIV strain for use in experimental investigations of the pathogenesis of epidemic VSV in livestock.

**Table 1:** Between- and within-group percent similarity among groups identified on phylogenetic analysis.

	**2019 Gp1**	**2019 Gp2**	**2019 Gp3**	**2020**
**2019 Gp1**	99.96%			
**2019 Gp2**	99.89%	99.96%		
**2019 Gp3**	99.84%	99.90%	99.93%	
**2020**	99.85%	99.81%	99.80%	99.95%

Within-group similarity is on the diagonal.

## Data availability statement

The datasets presented in this study can be found in online repositories. The names of the repository/repositories and accession number(s) can be found in the article/Supplementary material.

## Ethics statement

Ethical review and approval was not required for the animal study because all samples included in this study were collected as diagnostic samples by state and federal animal health officials or private veterinarians. Written informed consent for participation was not obtained from the owners because all samples included in this study were collected as diagnostic samples by state and federal animal health officials or private veterinarians.

## Author contributions

MB performed sequence assembly and genomic analysis and drafted the manuscript. CR and KR performed virus sequencing. LV-S performed genomic analysis. AP-M performed sampling activities. CM and LR conceived the study. All authors read and approved the final manuscript.

## Supplementary material

Supplementary Table S1 Metadata and accession numbers for 86 VSIV sequences collected in Colorado during the 2019 VSIV outbreak.
